# Patient satisfaction as a quality indicator in dermatological care: cross-sectional study in two tertiary institutions with residency programs^☆^

**DOI:** 10.1016/j.abd.2025.501228

**Published:** 2025-11-03

**Authors:** Manuel Gahona, Silvia Alejandra Prada, Daniela Chaparro

**Affiliations:** a Department of Dermatology, Hospital Universitario San Ignacio, Pontificia Universidad Javeriana, Bogotá, Colombia; b Department of Epidemiology, Universidad del Rosario, Bogotá, Colombia

**Keywords:** Health services Research, Internship and residency, Patient satisfaction, Quality of health care

## Abstract

**Background:**

Patient satisfaction is a key indicator of healthcare quality and plays a crucial role in strengthening the physician-patient relationship. In dermatology, it contributes to greater treatment adherence and improved clinical outcomes, particularly in chronic skin conditions. However, limited studies have comprehensively evaluated the factors influencing satisfaction in outpatient dermatological settings, especially in academic institutions.

**Objective:**

To identify factors associated with patient satisfaction during in-person dermatology visits at two tertiary care hospitals with postgraduate training programs and measure global satisfaction.

**Methods:**

A cross-sectional analytical study was conducted using a structured survey assessing six domains of care: physician interaction, verbal and non-verbal communication, consult timing, privacy, teaching activity, and infrastructure. A multivariate logistic regression model was used to determine variables associated with overall satisfaction, adjusting for potential confounders.

**Results:**

Most patients reported high satisfaction ([90.5%]), particularly with physician interaction. Key predictors of dissatisfaction included: inadequate bedside manner (Aor = 0.01 [95% CI 0.00–0.04], p < 0.001), discomfort during physical examination (aOR = 0.17 [95% CI 0.05–0.60], p = 0.006), presence of students during medical interview (aOR = 0.13 [95% CI 0.04–0.42], p < 0.001), and not use of white lab coat (aOR = 0.06 [95% CI 0.02–0.25], p < 0.001).

**Study limitations:**

The cross-sectional design prevents causal inference, and subjective responses may be influenced by social desirability bias. Context-specific findings may limit generalizability.

**Conclusions:**

Physician bedside manners, communication, and teaching dynamics significantly affect patient satisfaction. Targeted interventions in medical training and institutional policies may improve patient-centered care outcomes. Associations observed were statistically robust, minimizing the risk of spurious conclusions.

## Introduction

The World Health Organization (WHO) defines “quality” as the set of properties and characteristics that a system possesses to meet the needs of a population. Quality enables healthcare to achieve international health objectives.^1^ The Panamerican Health Organization states that health services must meet optimal standards of safety, effectiveness, timeliness, efficiency, and equitable access to provide high-quality care, with individuals, families, and communities at the center of this approach.^2^

Considering the patient as the ultimate purpose of health service delivery, it is imperative to analyze their perception of the quality of care received. For this reason, various studies have been conducted aiming to quantify user satisfaction through different surveys and assessment tools.^3^ This allows stakeholders within the health system to identify areas for improvement in order to increase user satisfaction levels, understanding satisfaction as an indirect measure of quality that influences clinical outcomes.^4^ In order to provide high-quality healthcare, the measurement of patient satisfaction must be carried out across all levels of care and medical specialties, including dermatology.^5^ Dermatology is a specialty with a high demand for private healthcare services; therefore, it is important to assess and analyze patient satisfaction. Dermatology is a clinical specialty with a significant care component in terms of providing both outpatient and inpatient services. Therefore, conducting studies that quantify patient satisfaction will allow for a better understanding of patient needs, identification of key factors when evaluating healthcare, and incorporation of this assessment into institutional processes.^6^ Patient satisfaction is also a factor that impacts clinical outcomes; in patients with chronic diseases who require prolonged treatments, it has been shown that higher satisfaction is associated with greater adherence to the prescribed treatments and recommendations.^4^

## Objective

### General objective

To measure the overall satisfaction of patients attending the dermatology service (in-person) at two healthcare institutions with postgraduate dermatology training in Bogotá during the period from November 1, 2024, to April 16, 2025.

### Specific objectives

1) To describe the sociodemographic characteristics of patients attended in the outpatient dermatology service (in-person) at two healthcare institutions in terms of age, gender, education level, residence, socioeconomic status, health insurance affiliation, type of consult, and recruitment site. 2) To describe the characteristics of the outpatient dermatology service points of care (in-person) at two healthcare institutions in terms of availability of restrooms and examination gowns in the doctor’s office, personnel present during care, and specialists’ attire. 3) To quantify the prevalence of overall satisfaction and the different domains of satisfaction among patients attended in the outpatient dermatology service (in-person) at two healthcare institutions in terms of interaction with the physician, verbal language, non-verbal language, waiting times, privacy, teaching activities, and infrastructure. 4) To identify the aspects with the highest and lowest satisfaction levels within the evaluated domains among patients attended in the outpatient dermatology service (in-person) at two healthcare institutions. 5) To compare sociodemographic characteristics, consult aspects, and evaluate domains according to patient satisfaction among those attended in the outpatient dermatology service (in-person) at two healthcare institutions. 6) To determine the association between sociodemographic characteristics, consultation aspects, and evaluated domains with the overall satisfaction level of patients attended in the outpatient dermatology service (in-person) at two healthcare institutions. 7) To propose strategies to improve patient satisfaction levels in the outpatient dermatology service (in-person) at two healthcare institutions.

## Methods

This is a cross-sectional observational study. The participating Institutions were private (Hospital Universitario San Ignacio) and public (Hospital Universitario La Samaritana) from Bogotá D.C, Colombia; the data collection period was from November 1, 2024, to April 16, 2025. All Patients who attended the outpatient dermatology service (in-person) were aimed to participate in the present investigation. The inclusion criteria were patients of any gender, aged over 18-years, and patients who had provided informed consent to participate in the questionnaire. The exclusion criteria were patients with cognitive disabilities, patients with visual impairments, patients with incomplete records (more than 20% missing data, i.e., 8 variables) or lacking information on the studied outcomes, patients with altered states of consciousness, and patients who completed the satisfaction survey but did not respond regarding consent to participate in the study. Given the descriptive nature of the study and the absence of a research hypothesis, no sample size calculation is required. Sampling will be convenience-based, inviting all eligible patients who meet the criteria to participate immediately after their in-person dermatology visit.

A survey was designed and implemented through a server running the Research Electronic Data Capture (REDCap) web application in the period between May 2024 and April 2025 to collect and store data virtually. At the end of the dermatology consult, the attending physician will invite the patient to participate in the study. Upon affirmative response, the physician will send the patient a link (or QR code) via email directing them to the questionnaire created in REDCap. Upon accessing the link, the first document the patient is requested to complete is the informed consent form; once consent is provided, the quality survey begins.

### Analysis plan

For descriptive analysis of qualitative variables, absolute frequencies and percentages will be used. For quantitative variables, measures of central tendency will be applied according to normality tests for each variable unless the sample size exceeds 30, in which case a normal distribution will be assumed, and the mean and standard deviation will be reported.

For bivariate analysis, two cohorts will be formed based on overall patient satisfaction: Satisfied (Overall satisfaction = Very satisfied or satisfied) and Not satisfied (Overall satisfaction = Neutral, Dissatisfied, Very dissatisfied). To analyze differences between groups for qualitative variables (gender, education, residence, socioeconomic status, health insurance affiliation, consult type, recruitment site, service point characteristics, and satisfaction domains), Chi-Square or Fisher’s exact test will be applied, depending on sample size adequacy. For quantitative variables (age, number of persons in the doctor’s office), Student’s *t*-test or Mann-Whitney *U* test will be used based on normality tests, unless normality is assumed due to sample size, in which case only Student’s *t*-test will be used.

To identify factors associated with overall satisfaction, a multivariate logistic regression model will be built, with the number of predictors depending on the number of events in each satisfaction category. For this analysis, satisfaction-related variables will be dichotomized as “Satisfied” (including Very satisfied or Satisfied responses) and “Not Satisfied” (Neutral, Dissatisfied, Very dissatisfied). Initially, univariate logistic regression will estimate crude Odds Ratios (ORs) for each variable. Variables meeting significance criteria will be selected as candidates for the multivariate model, which will be adjusted by backward selection.

A p-value less than 0.05 will be considered statistically significant. Analyses will be performed using R software (version 4.5.0) and RStudio.

### Ethical considerations

According to Resolution 8430 of 1993, this research is considered minimal risk as it involves only the completion of a survey.^7^ The ethical principles of respect, beneficence, justice, and protection of human dignity will be observed.

### Risks and benefits

To maintain participant confidentiality and ensure proper data handling, the following measures will be taken: data will be collected and stored on the REDCap platform, which offers a high level of security and is specifically designed for managing sensitive data in clinical and biomedical research. REDCap anonymizes the database by assigning a numeric code to each patient to protect confidentiality during statistical analysis. It also keeps a reversible coding record linking the survey responses to the informed consent to allow data validation if needed. Dissemination of study results will use anonymized data to protect patient confidentiality. This study was conducted after obtaining approval from the Institutional Ethics Committees of Hospital Universitario San Ignacio and Hospital Universitario La Samaritana. STROBE statement adherence points have been respected in the study design.

## Results

Data were collected from a total of 437 patients who attended in-person dermatology visits at the two participating institutions. The data are summarized in Table 1.

**Table 1:** Sociodemographic and clinical characteristics of the study population.

Characteristic	Overall, n (%)	Hospital Universitario de la Samaritana, n (%)	Hospital Universitario San Ignacio, n (%)	p-value
**Total (n)**	437	345	92	
**Age (years), mean (SD)**	45.02 (18.52)	45.35 (18.63)	43.78 (18.19)	0.472
**Male gender**	150 (34.3)	119 (34.5)	31 (33.7)	0.984
**Educational level**				0.303
None	6 (1.4)	5 (1.4)	1 (1.1)	
Elementary School	56 (12.8)	49 (14.2)	7 (7.6)	
High School	137 (31.4)	112 (32.5)	25 (27.2)	
Technical	62 (14.2)	49 (14.2)	13 (14.1)	
Higher Education	127 (29.1)	93 (27.0)	34 (37.0)	
Postgraduate studies	49 (11.2)	37 (10.7)	12 (13.0)	
**Urban residence**	369 (84.4)	284 (82.3)	85 (92.4)	0.027
**Socioeconomic stratum**				
Stratum 1	66 (15.1)	65 (18.8)	1 (1.1)	
Stratum 2	104 (23.8)	90 (26.1)	14 (15.2)	
Stratum 3	179 (41.0)	150 (43.5)	29 (31.5)	
Stratum 4	72 (16.5)	36 (10.4)	36 (39.1)	
Stratum 5	16 (3.7)	4 (1.2)	12 (13.0)	
**Health insurance scheme**				0.011
Contributory	257 (58.8)	197 (57.1)	60 (65.2)	
Subsidized	81 (18.5)	73 (21.2)	8 (8.7)	
Private	63 (14.4)	44 (12.8)	19 (20.7)	
Special regimen	36 (8.2)	31 (9.0)	5 (5.4)	
**Type of consult**				0.149
First-time	249 (57.0)	203 (58.8)	46 (50.0)	
Follow-up	168 (38.4)	129 (37.4)	39 (42.4)	
Procedure	20 (4.6)	13 (3.8)	7 (7.6)	

The average age was 45.02 years (SD = 18.52), and 34.3% (150) were male. The most frequent educational level was secondary education (137 [31.4%]), followed by higher education (127 [29.1%]) and technical studies (62 [14.2%]). Approximately 40% (176) of the patients had postgraduate studies, and only a small percentage of patients didn’t receive any kind of education (6 [1.4%]). Most patients lived in urban areas (369 [84.4%]) in socioeconomic level 1, 2 and 3 (349 [79.9%]).

In terms of overall satisfaction, 90.5% (395) of patients were satisfied with the care received (Very satisfied = 297 [68%], Satisfied = 99 [22.7%]). A small proportion of patients were neutral or dissatisfied with the care: 10 [2.3%] and 2 [0.5%], respectively. Only 6.6% (29) reported a high level of dissatisfaction.

The evaluated domains included physician interaction, verbal and nonverbal communication, consultant times, privacy, teaching activity, and infrastructure.

Patients were highly satisfied with the following aspects:

Domain 1: Medical history-taking (313 [71.6%]) – closely followed by physician behavior and physical examination;

Domain 2: Communication with the billing department (296 [67.7%]);

Domain 3: Physician wearing a white lab coat (272 [62.2%]);

Domain 4: Duration of the consult (246 [56.3%]);

Domain 5: Privacy during the consult (252 [57.7%]);

Domain 6: Presence of students during the consult (216 [49.4%]);

Domain 7: Comfort in the waiting room (223 [51%]).

Across the entire study, the item with the highest level of satisfaction was medical history-taking (313 [71.6%]), whereas the lowest was the taking of clinical photographs (190 [43.5%]).

### Factors associated with patient satisfaction

Patients were divided in two groups according to satisfaction: Satisfaction group = 396 (Very satisfied and Satisfied) vs. No satisfaction group = 41 (Neutral, Unsatisfied, Highly unsatisfied). There were no differences in sociodemographic characteristics between groups, but there were statistically significant differences (p < 0.001) across all the domains. In the satisfied group, the aspects with the highest proportion of high satisfaction were physician behavior (336 [75.5%]), medical history-taking (333 [75.3%]), and physical examination (323 [72.7%]). Among unsatisfied patients, the highest satisfaction was related to the physician's self-introduction with name and title (17 [41.5%]), while the highest levels of dissatisfaction were related to bedside manner, physical examination, and medical interview. Approximately one-third of patients in this group reported high dissatisfaction with the physician's tone of voice (2 [4.1%]), not use of a white lab coat (14 [34.1%]), body language (13 [31.7%]), and the appointment scheduling process (13 [31.7%]). Less than half of the group (190 [43.5%] were highly satisfied with the clinical photographs, waiting room comfort, and the presence of students during medical interview (Table 2).

**Table 2:** Highest- and lowest-rated aspects across domains.

Groups	Aspects with Highest Satisfaction (Very Satisfied)	Aspects with Lowest High Satisfaction	Aspects with Highest Dissatisfaction
Satisfied (n = 396)	Physician behavior (75.5%)	Interruptions during the consultation (50.8%)	Presence of students during consult (2.3%)
	Medical history-taking (75.3%)	Doctor’s office temperature (47.5%)	Interruptions during the consultation (1.3%)
	Physical examination (72.7%)	Clinical photographs (46.2%)	Presence of students during history-taking (1.3%)

Not satisfied (n = 41)	Introduction with name and title (41.5%)	Clinical photographs (17.1%)	Physician Behavior (48.8%)
	Treatment explanation (41.5%)	Comfort of the waiting room (17.1%)	Physical examination (43.9%)
	Medical history-taking (41.5%)	Presence of students during history-taking (14.6%)	Medical history-taking (41.5%)
	Explanation of the disease (39%)		

In the univariate logistic regression analysis, all variables were associated with overall satisfaction (p < 0.05). The final model (adjusted using backward selection) retained the variables with the strongest associations and clinical relevance.

After adjusting for the variables with the highest statistical significance, unsatisfactory bedside manner and the presence of students during medical interview decreased the likelihood of overall satisfaction (aOR = 0.01 [95% CI 0.00–0.04], p < 0.001; aOR = 0.13 [95% CI 0.04–0.42], p < 0.001, respectively). Likewise, specialists not using a white lab coat reduced the probability of satisfaction (aOR = 0.06 [95% CI 0.02–0.25], p < 0.001).

In conclusion, the probability of overall satisfaction was 39.82% when there was appropriate bedside manner, comfort during the physical examination, no students present in the doctor’s office, and the specialist wore a medical gown (Table 3).

**Table 3:** Factors most strongly associated with patient satisfaction.

Variable	Crude OR	[95% CI]	p-value	Adjusted OR	[95% CI]	p-value
Intercept				39.82	21.92–82.22	<0.001^a^
Unsatisfactory bedside manner	0.01	0.00–0.03	<0.001^a^	0.01	0.00–0.04	<0.001^a^
Discomfort during physical examination	0.25	0.12–0.52	<0.001^a^	25.76	4.46–190.04	0.001^a^
Presence of students during the interview	0.11	0.05–0.21	<0.001^a^	0.13	0.04–0.42	<0.001^a^
Specialist not wearing a white lab coat	0.05	0.02–0.11	<0.001^a^	0.06	0.02–0.25	<0.001^a^

Model fit: AIC = 152.8, *R*^2^ = 0.52, AUC = 0.897, Maximum VIF = 2.46.

a Significant p-value < 0.05.

The model showed good explanatory power (*R*^2^ = 0.52). The Hosmer-Lemeshow goodness-of-fit test could not be adequately calculated, possibly due to limitations in the number of observations.

## Discussion

Measuring patient satisfaction in healthcare systems has become an important indicator of service quality, based on the premise that healthcare systems should be patient-centered.^6^

In dermatology, patient satisfaction plays an important role in the doctor-patient relationship, as it increases the likelihood of patient return and reduces claims of medical negligence and malpractice errors. Additionally, services with high satisfaction levels tend to be more productive, which positively impacts clinical outcomes by improving therapeutic adherence among patients with chronic skin diseases. Patient satisfaction is also an important factor when seeking international quality accreditations, such as The Joint Commission on Accreditation of Healthcare Organizations.^4^

Various international studies have been conducted to evaluate patient satisfaction levels in dermatology consult, both in-person and via telemedicine, encompassing clinical and cosmetic dermatology.^6^, ^8^, ^9^, ^10^, ^11^, ^12^, ^13^, ^14^, ^15^

Below are the results of the present study compared with the existing literature, along with a list of recommendations for each evaluated domain.

### Medical interaction

Bedside manner was one of the best-rated aspects. Dissatisfaction with bedside manner (even when the patient is comfortable during physical exam, there is no students present, and the physician wears a white lab coat) reduced the likelihood of overall satisfaction by 99% (aOR = 0.01 [95% CI 0.00–0.04], p < 0.001). It has already been established that respect, empathy, and effective communication between physician and patient are central elements in building patient satisfaction.^16^, ^17^

On the other hand, negative communication in the hospital environment, manifested as Rudeness, Disdain, and Aggression (RDA), is a recognized problem in healthcare systems that increases levels of stress, depression, and the intention to leave the profession among healthcare workers. It has also been associated with worse clinical outcomes, including mortality.^18^ Approximately 40% of physicians report that RDA significantly affects their workday, and 7% commit clinical errors for this reason, which can negatively impact patient satisfaction.^16^

Disrespectful behavior reduces the likelihood that patients will voice their concerns, ask questions, or even share relevant information. Poor communication skills reduce the probability that patients will understand the information and feel satisfied with the bedside manner.^17^

The hospital environment also affects physician behavior, especially since there is an institutional tendency to ignore problems of frustration, poor communication, and disrespect among healthcare professionals.^18^, ^19^ These issues often occur in high-stress settings that can increase individual feelings of anxiety, insecurity, depression, aggression, narcissism, and even trigger a “survival mode” response that reduces empathy.^16^

#### Recommendations

• Establish an interdisciplinary committee (including administrative staff, physicians with varying levels of experience, and nursing personnel) to thoroughly analyze and clearly define disrespectful behavior among institutional staff and toward patients. This committee should provide a list of practical examples to help identify such behavior and specify a course of action when it is detected. A zero-tolerance policy should be implemented, with interventions ranging from educational programs to disciplinary measures, accompanied by a non-retaliation policy to protect those who report misconduct. Evidence shows that these types of institutional policies increase the effectiveness of addressing these issues.^20^
• Create a professional code of conduct that clearly defines the expected behavior for every member of the institution, to improve quality outcomes (without assuming that staff already know these expectations). Behavioral changes within a community often occur when a principle is collectively accepted as an absolute truth, for example, “Absolutely everyone deserves respect”.^21^
• Training in “agenda-setting”; this approach is based on allowing the patient to initially express all their concerns and then prioritizing and negotiating with them the most relevant issues to address during the consult or later if necessary.

### Verbal and nonverbal communication

Voice tone had the lowest percentage of high satisfaction (214 [49%]), and speech speed, although minimal, was the most dissatisfying component in this domain (17 [3.9%]). Previous studies have reported that physicians who speak quickly may convey more information, demonstrate attentiveness and care toward the patient, and even appear more persuasive, which is generally perceived as a positive factor in patient satisfaction (p < 0.01). It has also been shown that high-pitched tones reduce patient satisfaction (p < 0.001). However, both tone and speech speed become less relevant when the physician possesses strong professional credentials (degrees, academic training, recognized employment, etc.).^21^ Dissatisfaction with the professional's self-introduction with name and title was zero, compared to 66% (289) of patients who reported being highly satisfied in this area.

Non-verbal language was rated positively (272 [62.2%]). This aspect is positively associated with patients' sense of well-being, self-esteem, and social processes, even in the absence of other comfort strategies.^22^ Gestures such as nodding, maintaining eye contact, eyebrow movements, as well as the amount and location of physical contact, also influence patient satisfaction. However, in the present study, only the absence of a white lab coat had a significant impact on satisfaction, reducing its likelihood by 93.7% (aOR = 0.06 [95% CI 0.02–0.25], p < 0.001).^23^

#### Recommendations

• The physicians should introduce themselves by name and title to communicate their professional credentials, maintain a moderate-to-fast speaking pace, and avoid unnecessarily raising their voice when speaking with patients.
• It is advised to consistently wear a white lab coat during consult and to use gestures such as a light touch on the shoulder, forearm, or hand, or a handshake when greeting patients.

### Consult timing

There was an adequate level of satisfaction with the 20-minute consult (246 [56.3%]). Previous studies have reported satisfaction rates as high as 74% with consults lasting as little as 12.1 minutes (p < 0.001, *r* = 0.38), with a 0.04-point increase in satisfaction for each additional minute of consult time (p = 0.001 [95% CI 0.01–0.06]).^24^ A positive association has also been found in consultation lasting 9.85 ± 0.37 minutes (p = 0.005).

Efficient administrative processes ‒ such as fast appointment scheduling, quick billing procedures, and effective communication with administrative staff ‒ have been shown to increase patient satisfaction by 3.58 times (aOR = 3.59 [95% CI 2.36–5.42], p < 0.001).^25^ Although waiting time typically impacts overall satisfaction, in this study population, it was not statistically significant. Despite average waiting times of up to 64 minutes, 57% of patients reported being satisfied (p = 0.001).

#### Recommendations

• Physicians should maintain the current consult duration (20-minutes) and ensure punctuality.
• The institution must provide the necessary tools and staff to facilitate appointment scheduling and billing procedures. Additionally, it should ensure effective communication between different teams (both medical and non-medical) to streamline care processes.

### Privacy

Less than 50% (190) of patients reported being satisfied with privacy during the medical photographs. In the literature, most patients (79.2%) report satisfaction with medical photography, and many express contentment in contributing to research, particularly when institutional cameras are used. Taking photos with smartphones may cause distrust and be perceived as unprofessional, although one study reported a 79% acceptance rate for smartphone photography.^26^

Privacy can influence a patient's decision to allow physical examination or to share relevant information. In one study, 10% of patients declined a physical examination due to privacy concerns; closing doors or providing curtained areas increased the perceived privacy from 21% to 89%.^27^

#### Recommendations

• Always close the door or use curtains to ensure patient privacy.
• Always use informed consent to take medical pictures, clearly explain to the patient the measures taken to ensure the security and confidentiality of the pictures, and communicate the purpose and academic value of the photographs.
• Whenever possible, use an institutional camera for a photograph.

### Teaching activity

The participation of students during the clinical interview decreased the likelihood of overall patient satisfaction by 87% (aOR = 0.13 [95% CI 0.04–0.42], p < 0.001). In the review by Mol et al. (2011), the presence of students did not affect patient satisfaction and was actually associated with benefits such as longer consult times, more thorough examinations, and improved patient education.^28^ Other studies have shown that patients report greater satisfaction when students are present, as they feel altruistic and believe they are contributing to the students’ education.^28^

Since the presence of students can limit a patient's willingness to share intimate or emotional issues, this may have influenced these results, particularly considering that many dermatological conditions are associated with feelings of stigmatization or embarrassment, often related to the anatomical location of the lesions.^27^

#### Recommendations

• Always ask the patient, before starting the consult, whether they agree with the participation of students.
• Even if the patient authorizes student involvement, it may be advisable to ask the students to step out of the consult to allow the patient to discuss any intimate or emotional aspects.

### Infrastructure

Paradoxically, discomfort during the physical examination was associated with a higher probability of overall satisfaction (aOR = 25.76 [95% CI 4.46–190.04], p = 0.001). Although this association may reflect a real phenomenon ‒ for example, that patients perceive thorough, uncomfortable examinations as indicators of careful medical attention ‒ it could also result from reporting bias or the limitations of sample size. Another possibility is that under conditions involving respectful bedside manner, presence of a physician wearing a white lab coat, and absence of students, discomfort becomes less relevant.

In patients with disabilities, studies have shown that 27% are not examined on the doctor’s office table, which is associated with a lower likelihood of rating the physician’s care and work positively (59% and 39%, respectively).^29^

The size of the doctor’s office is related to the patient’s sense of privacy; small spaces where patients can overhear other conversations reduce satisfaction from 36% to 14%.

#### Recommendations

• At an institutional level, it is important to ensure that doctors’ offices are either sufficiently spacious or provide adequate privacy so that clinical interactions cannot be overheard outside.

### Study limitations

An important strength of this study is that it evaluates a wide variety of aspects related to healthcare, providing a comprehensive context to understand this phenomenon. Additionally, the parameters of the multivariate regression model were adequate, suggesting that the variables used can discriminate between satisfied and dissatisfied patients.

However, several limitations must be considered, such as the cross-sectional nature of the study, which prevents establishing causal relationships. It is important to note that the measurement of satisfaction is subject to social biases, and the factors impacting it vary considerably across populations. For this reason, unusual outcomes, such as discomfort during physical exam, require verification through additional analyses, ideally using hierarchical models or sensitivity analyses.

Another limitation common to all studies evaluating satisfaction is the complexity of analyzing all possible factors related to satisfaction and appropriately measuring them to avoid collinearity issues. It is important to include aspects related to technology (virtual appointment scheduling, scheduling methods, preferred contact methods, use of medical instruments during consult, etc.), which are increasingly common in medical practice.

Analytical studies are recommended to assess the direction of causal relationships, as well as qualitative analyses to explore patients' subjective perceptions in depth, and even more so, experimental evaluations of educational interventions aimed at improving the relational competencies of physicians and students.

## Conclusions

This study offers a comprehensive evaluation of patient satisfaction during dermatological consultations, examining various domains such as physician-patient interaction, verbal and non-verbal communication, waiting times, privacy, teaching activities, and infrastructure. Most patients reported high overall satisfaction with the medical interview, physician’s behavior, and physical examination.

Statistical analysis showed significant differences between groups in all domains and identified key factors affecting patient satisfaction. Notably, unsatisfactory physician behavior, discomfort during the physical exam, the presence of students during the interview, and the lack of a white lab coat worn by the physician significantly decreased the likelihood of overall satisfaction.

The multivariate regression model demonstrated moderate explanatory power, supporting the clinical and statistical importance of the variables studied. However, limitations related to the cross-sectional design and the complexity of measuring satisfaction accurately ‒ due to possible social biases and population differences ‒ are recognized.

This study emphasizes the need for further analytical, qualitative, and experimental research to better understand patient perceptions, establish causal relationships, and evaluate interventions aimed at improving the relational skills of healthcare providers and medical students.

## ORCID ID

Silvia Alejandra Prada: 0000-0002-3256-0331

Daniela Chaparro: 0000-0002-8848-7882

## Financial support

This research did not receive any specific grant from funding agencies in the public, commercial, or not-for-profit sectors.

## Authors' contributions

Manuel Gahona: Conceptualization; Data curation; Resources; Writing-original draft.

Silvia Alejandra Prada: Conceptualization; Resources; Formal analysis; Writing-original draft.

Daniela Chaparro: Conceptualization; Data curation; Writing-original draft.

## Research data availability

The entire dataset supporting the results of this study was published in this article.

## Conflicts of interest

None declared.
